# Tobacco smoking and vascular biology and function: evidence from human studies

**DOI:** 10.1007/s00424-023-02805-z

**Published:** 2023-03-24

**Authors:** Omar Hahad, Marin Kuntic, Ivana Kuntic, Andreas Daiber, Thomas Münzel

**Affiliations:** 1 Department of Cardiology – Cardiology I, University Medical Center of the Johannes Gutenberg University Mainz, Langenbeckstraße 1, 55131 Mainz, Germany; 2 German Center for Cardiovascular Research (DZHK), partner site Rhine-Main, Mainz, Germany

**Keywords:** Tobacco cigarette smoking, Endothelial dysfunction, Inflammation, Oxidative stress, Human studies

## Abstract

Tobacco cigarette smoking is among the most complex and least understood health risk factors. A deeper insight into the pathophysiological actions of smoking exposure is of special importance as smoking is a major cause of chronic non-communicable diseases, in particular of cardiovascular disease as well as risk factors such as atherosclerosis and arterial hypertension. It is well known that smoking exerts its negative effects on cardiovascular health through various interdependent pathophysiological actions including hemodynamic and autonomic alterations, oxidative stress, inflammation, endothelial dysfunction, thrombosis, and hyperlipidemia. Importantly, impaired vascular endothelial function is acknowledged as an early key event in the initiation and progression of smoking-induced atherosclerosis. Increasing evidence from human studies indicates that cigarette smoke exposure associates with a pathological state of the vascular endothelium mainly characterized by reduced vascular nitric oxide bioavailability due to increased vascular superoxide production. In the present overview, we provide compact evidence on the effects of tobacco cigarette smoke exposure on vascular biology and function in humans centered on main drivers of adverse cardiovascular effects including endothelial dysfunction, inflammation, and oxidative stress.

## Introduction

Today, tobacco cigarette smoking is a known risk factor for many cardiovascular diseases (CVD) [56]. The World Health Organization (WHO) estimates that 20 % of all deaths from coronary heart disease are related to cigarette smoking [79].

In 1950s, the link between tobacco smoking and lung cancer was established, but it was still not clear which components of smoke were mainly tumorigenic. It was later discovered that tobacco-specific nitrosamines were the main carcinogenic compound of cigarette smoke [45]. CVD are one of the most complex group of diseases, as they have a multifaceted pathomechanism and are subject to a variety of risk factors and genetic predispositions [15], which makes it hard to study chemical toxicity to the cardiovascular system mechanistically. Tobacco cigarette smoke is containing more than 9000 different identified chemicals [66], but only a handful of them have really been correlated significantly to CVD on a mechanistical level. For instance, the study of Fowles et al. pointed out that due to the lack of toxicological data, only two chemical compounds, hydrogen cyanide and arsenic, could be perceived as substantial cardiovascular risk factors [21]. These findings presented a challenge for researchers and medical professionals to fight against both cigarette smoking and CVD, as the clear identification of the main toxins in cigarette smoke triggering CVD is still lacking.

## Endothelial dysfunction

The first studies on direct effects of smoking on endothelial function, that yielded potential mechanistical explanations, were done in the early 1990s. Celermajer et al. demonstrated that tobacco cigarette smoking was associated with a reduced flow-mediated dilation (FMD) [10]. The reduction of FMD caused by smoking was used as a marker for endothelial dysfunction (ED), thus demonstrating for the first time a clinically relevant link between tobacco smoke and vascular dysfunction.

Although the study by Celermajer et al. was observational in nature, many other studies confirmed the observation. One of the earliest studies to offer experimental evidence for reduced FMD in smokers demonstrated that FMD was significantly reduced after smoking only one tobacco cigarette in a controlled environment [48]. The authors further demonstrated that, in contrast to FMD, nitrate-induced endothelium-independent vasodilation was not impaired. Likewise, in healthy smokers, smoking only one tobacco cigarette was sufficient to increase systolic and diastolic blood pressure, forearm resistance, and carotid wall tension [4]. Studies done on isolated human middle cerebral arteries showed that after exposure to soluble particles from cigarette smoke, acetylcholine (ACh)-induced endothelium-dependent relaxation was reduced [83]. No decrease in relaxation was observed after administration of the same concentration of pure nicotine. On the other hand, a study in healthy individuals showed that pure nicotine infusion did show a selective impairment of endothelium-dependent vascular relaxation, whereas the endothelium-independent vasodilation was not affected [13]. Acute cigarette smoking and nicotine chewing gum consumption impaired endothelium-dependent, but again not the endothelium-independent relaxation of the brachial artery [68]. This indicates that nicotine is playing an important role in smoking-induced ED, although the effects are mainly considered to be acute. Passive smoking also caused ED in a cohort study [12] and in an experimental setting, where patients had a reduced coronary flow velocity reserve, a marker of ED [60].

ED is usually characterized by the impairment in the chemical signaling between the endothelial cells and smooth muscle cells [56]. One of the most important signaling pathways is the nitric oxide (^•^NO) signaling. ^•^NO is produced by the endothelial nitric oxide synthase (eNOS) and diffuses to the smooth muscle cells where it binds to the soluble guanylyl cyclase (sGC) that activates the cGMP-dependent protein kinase [63], leading to a reduction of intracellular calcium levels and thus to relaxation [53]. Reduced vascular bioavailability of ^•^NO was established in healthy smokers [43]. The low bioavailability of ^•^NO is at least in part the result of uncoupling of eNOS, which inhibits production of ^•^NO, and the scavenging of ^•^NO by the superoxide radical leading to the formation of the highly reactive peroxynitrite (ONOO-) [5, 72]. eNOS becomes uncoupled when its cofactor tetrahydrobiopterin (BH4) is oxidized by ONOO^-^ into the BH_3_^.^ radical [54]. The importance of this mechanism was highlighted by studies where supplementation with BH_4_ improved endothelium-dependent vasorelaxation in chronic smokers [37] and in isolated blood vessels [38].

**Fig. 1:**
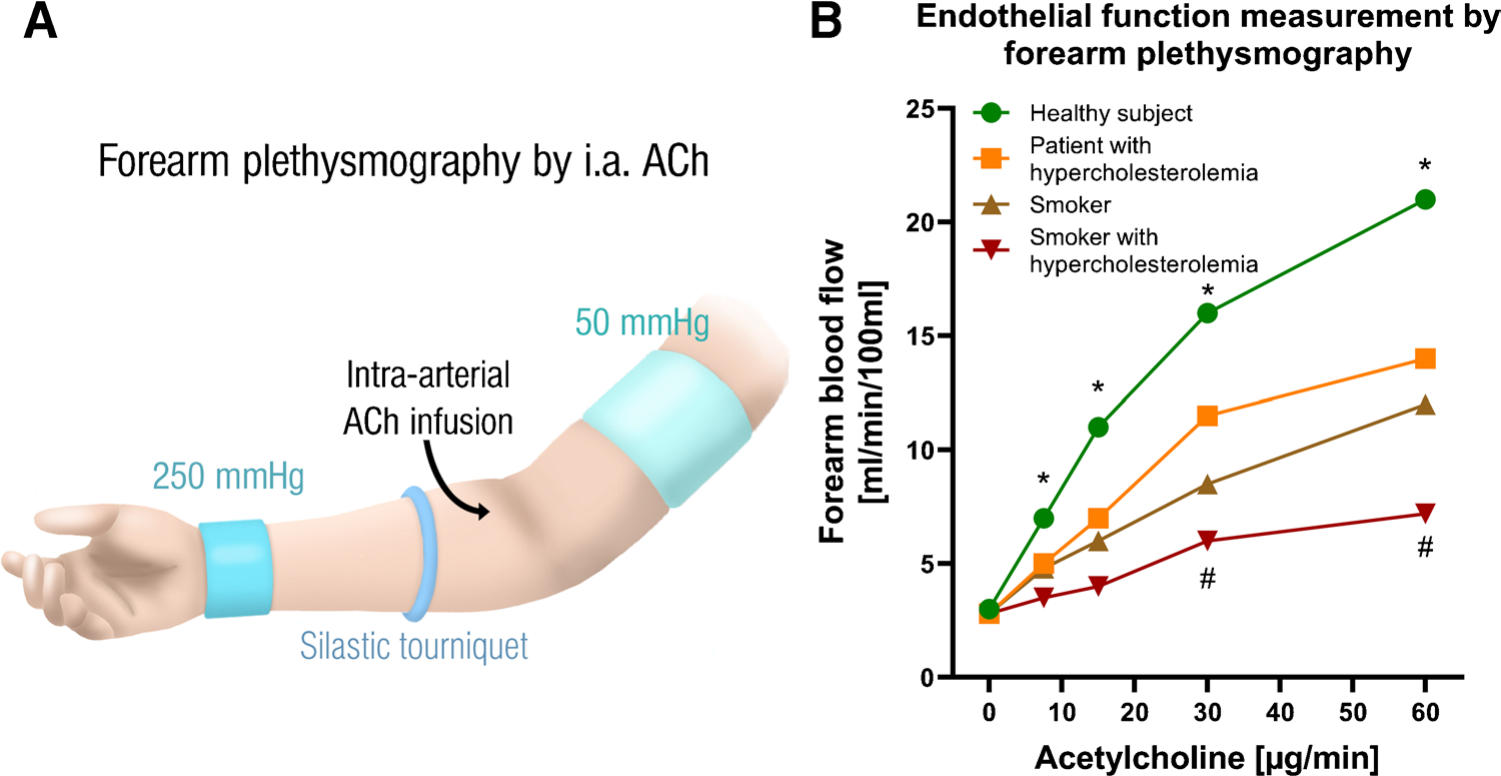
Method of measuring endothelial function and effects of tobacco smoking on endothelial function in subjects with or without hyperlipidemia. **A** The acetylcholine (ACh)-dependent vasoreactivity of a forearm conduction vessel after intra-arterial ACh infusion can be used to determine endothelial function, i.e., vessel dilatation or increase in blood flow, in humans (plethysmography of the forearm). Vasodilation is measured by Doppler ultrasound after each cumulative dose of ACh, either by vasodilatation or by an increase in blood flow. Translated and used from references [ 85. ] with permission. Copyright © 2016 The British Pharmacological Society. **B** Curves for an increase in blood flow as a function of ACh dose in healthy subjects, hypercholesterolemic patients, long-term smokers, and hypercholesterolemic patients who are also smokers. All 3 patient groups have a significantly impaired endothelial function compared to the healthy subjects (shown by the lower increase in blood flow due to ACh). Asterisk (*) shows significant differences from starting point; hash (#) indicates significant differences versus healthy subjects (*P*<0.05). Values estimated and traced from reference [36] with permission Copyright © 1996, Wolters Kluwer Health

While acute tobacco smoking may cause ED in healthy subjects, clinical studies were also able to demonstrate additive negative effects of tobacco smoking and other pre-existing cardiovascular risk factors (such as hyperlipidemia) on ED [36]. Forearm plethysmography was used to assess endothelial function of forearm resistance vessels (Fig. 1). The infusion of intra-arterial ACh showed a reduced endothelial function in subjects with increased LDL levels and in smokers (20 pack-years). As shown in Fig. 1B, smokers have a partial deterioration of endothelial function, with a similar severity as in patients with hypercholesterolemia, with the presence of both risk factors (hypercholesterolemia and smoking) leading to a significant and severe deterioration of endothelial function [36]. A similar additive effect on ED was shown in the ALSPAC study (*n*=1266 teenagers) for the combined consumption of tobacco cigarettes and alcohol during adolescence [14].

**Fig. 2:**
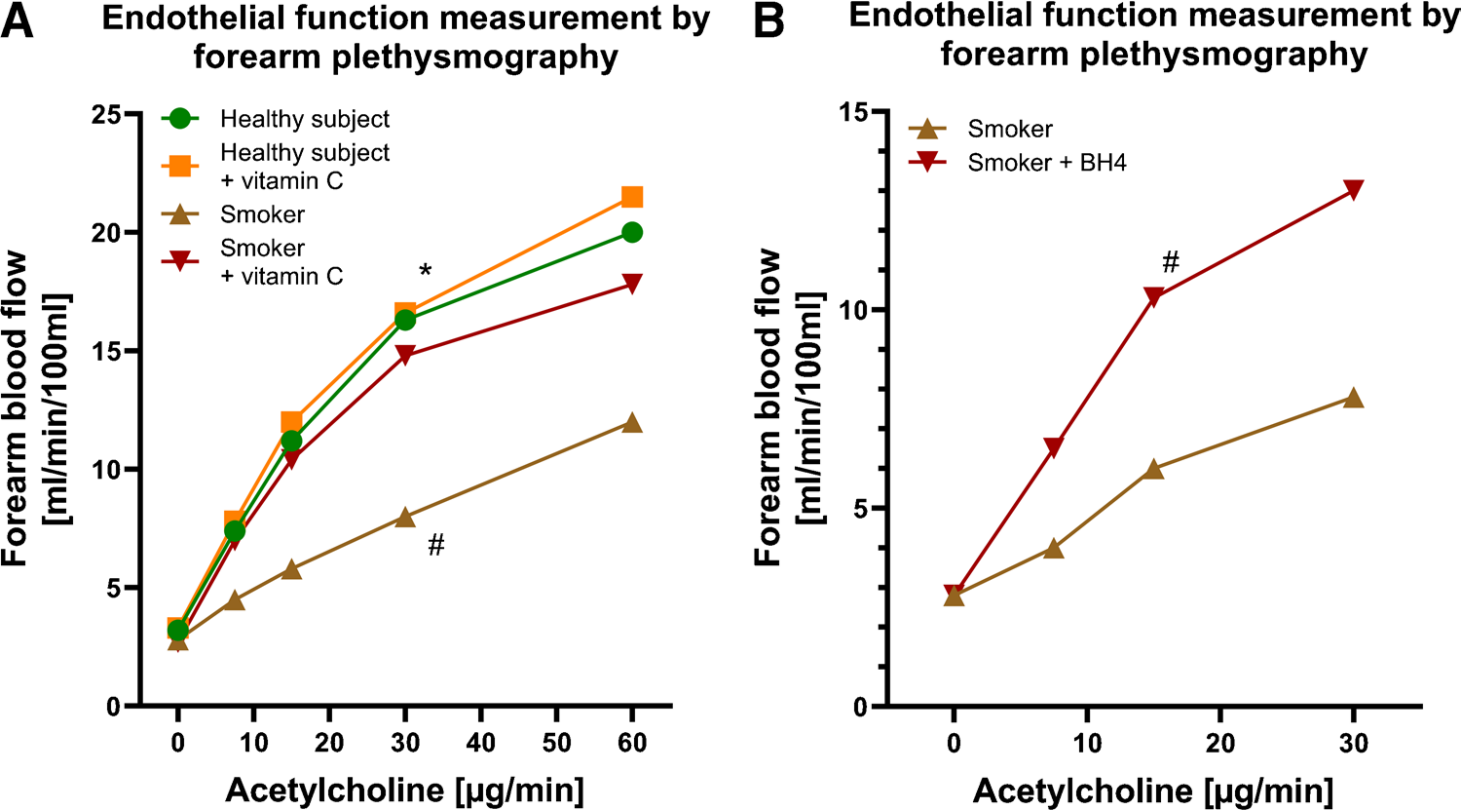
Effects of tobacco smoking on endothelial function (using forearm plethysmography) in volunteers and antioxidant interventions. **A** Endothelial function in tobacco smokers, i.e., the vasodilator capacity in response to intraarterial infusion of the endothelium-dependent vasodilator acetylcholine (ACh), was strikingly reduced in chronic smokers (history of more than 20 pack-years) and was restored to normal levels by the administration of the antioxidant vitamin C, compatible with a high degree of oxidative stress in resistance vessels of chronic smokers. In contrast, the administration of vitamin C had no effect in the healthy volunteers. Asterisk (*) shows significant differences from starting point; hash (#) indicates significant differences versus healthy subjects (*P*<0.05). Values estimated and traced from reference [35] with permission. Copyright © 1996, Wolters Kluwer Health. **B** Administration of tetrahydrobiopterin (BH4), an essential cofactor of the enzyme endothelial nitric oxide synthase, was able to normalize the impaired endothelial function in tobacco smokers. In the BH4 group of smokers, the ACh-induced increase in blood flow was significantly better than in placebo-treated smokers. Hash (#) indicates significant differences versus smokers (*P*<0.05). Values estimated and traced from reference [37] with permission. Copyright © 2000, Wolters Kluwer Health

The formation of reactive oxygen species (ROS=oxidative stress), inflammatory reactions, and ED have been established as central pathomechanisms of vascular damage and the development of cardiovascular disease from tobacco smoke. Smoking-induced ED was corrected by the antioxidant vitamin C indicating an involvement of ROS such as superoxide in causing this phenomenon [11,35] (Fig. 2A). As significant superoxide source, an uncoupled nitric oxide synthase was identified since a normalization of vascular dysfunction was achieved by administrating the eNOS cofactor tetrahydrobiopterin (BH_4_) [37] (Fig. 2B).

**Fig. 3:**
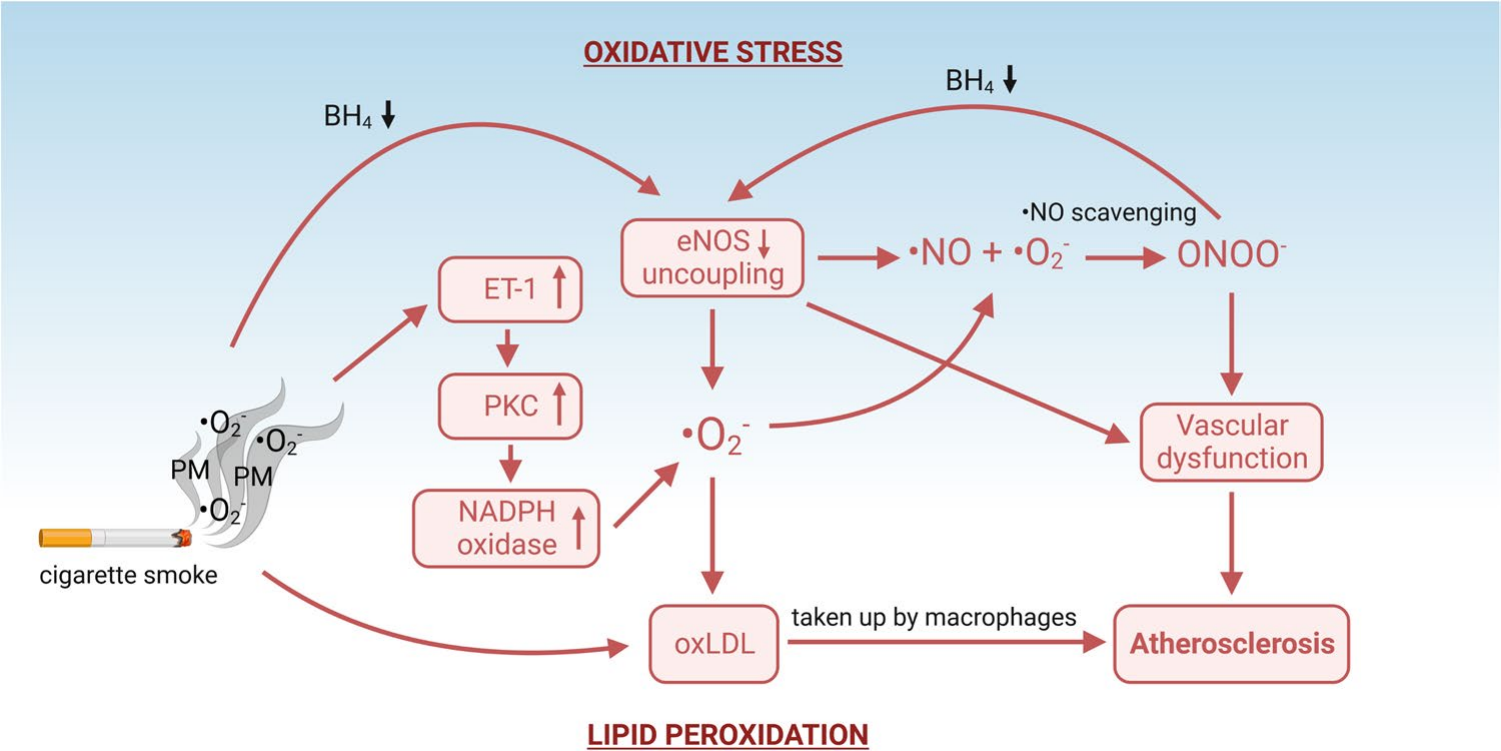
Proatherosclerotic molecular mechanisms related to vascular dysfunction. Oxidative stress can be both exogenous (from free radicals in tobacco smoke) and endogenous (from activation of free radical generating enzymes). Superoxide radical (O_2_^-^) can originate directly from cigarette smoke or be produced by uncoupled eNOS and NADPH oxidase. NADPH oxidase can be activated by ET-1 through stimulation of PKC, a kinase that activates the subunits of NADPH oxidase, causing it to become assembled and produce superoxide. Superoxide can directly scavenge •NO by forming peroxynitrite (ONOO^-^) or it can oxidize lipids, such as LDL. This mechanism not only impairs •NO signaling for vasodilation, but also promotes oxidized LDL accumulation in the infiltrated macrophages, propagating atherosclerosis. Created with BioRender.com

Another prominent factor that influences the pro-constrictive environment and endothelial function of the blood vessels found in smokers is endothelin-1 (ET-1) [8]. ET-1 is a potent vasoconstricting peptide that is approximately equipotent to angiotensin-2 or norepinephrine [82]. It is generally observed that smokers have higher circulating ET-1 concentrations [8, 28, 58]. A study done on smokers revealed that just after smoking three cigarettes, ET-1 induced a much greater vasoconstriction response than before smoking [43]. In addition, smoking only one cigarette resulted in an immediate increase in plasma ET-1 levels, which subsided after 15 min [26, 29]. ET-1 reduces ^•^NO bioavailability and influences endothelial function directly by both reducing eNOS expression [64, 73] and activity, and by activating the nicotinamide adenine dinucleotide phosphate (NADPH) oxidase, a superoxide producing enzyme located in vascular cells and inflammatory cells such as macrophages [2, 67, 84]. It was also observed that ET-1 is involved in activation of protein kinase C (PKC), which phosphorylates subunits of the NADPH oxidase complex to create the active, superoxide producing, state [46]. Interestingly, acute exercise also causes increase in circulating ET-1 levels [49], but chronic exercise reduces ET-1 and increases NO^.^ levels [50]. Smokers who exercise have better peripheral blood flow [3], but chronic increase in ET-1 can limit both exercise-induced vasodilation and blood flow in muscles [19]. A schematic overview of the molecular mechanisms relating to ED is presented in Fig. 3.

## Oxidative stress

As mentioned above, ^•^NO signaling is sensitive to oxidation by free radicals, making oxidative stress one of the most detrimental conditions for causing vascular dysfunction. Tobacco cigarette smoke not only increases vascular oxidative stress, but also contains free radicals itself, thus causing ED both by endogenous and exogenous sources [62]. Scavenging of ^•^NO by the superoxide radical to generate peroxynitrite is one of the most important mechanisms for vascular dysfunction. Superoxide radical can originate directly from tobacco cigarette smoke [62], or from activation of NADPH oxidase and uncoupling of eNOS [71]. Many studies demonstrated that free radical scavenging may have a beneficial effect on vascular oxidative stress. A study examining urinary levels of 8-epi-prostaglandin (PG) F2α, a stable product of lipid peroxidation, showed that administration of vitamin C and E decreased lipid peroxidation in chronic smokers [65]. As mentioned already, vitamin C markedly improved forearm blood flow in response to the endothelium-dependent vasodilator ACh in chronic smokers (Fig. 2) [35]. eNOS uncoupling and downregulation of the enzyme were also established in vivo and in vitro [1, 37, 72]. When BH_4_ is oxidized, eNOS uncoupling is equal with a reduction of ^•^NO production and starting the production of a superoxide radical instead, scavenging more ^•^NO and oxidizing more BH_4_, thus propagating the vicious cycle [42]. Thus, eNOS can be considered as an enzyme with two faces [20]. In an interesting study, authors have exposed human coronary artery endothelial cells to serum obtained from either chronic smokers or non-smokers, and found that eNOS activity and ^•^NO production were reduced, compatible with increased oxidative stress [6]. An increase in NADPH oxidase activity was also observed by measuring serum levels of soluble NOX2-derived peptide after smoking only one tobacco cigarette [9]. NADPH oxidase activation was confirmed in vitro [39] and in vivo [30].

Cigarette smoking will also interfere with the antioxidant defense system. A study observed that heavy smokers have higher circulating markers of lipid peroxidation (malondialdehyde) and impaired antioxidant system, envisaged by the changes in activity of glutathione peroxidase and glutathione reductase [70]. Reduction in blood cell superoxide dismutase (SOD) was also observed in smokers [59]. On the other hand, increased levels of SOD were observed in smokers, which could be the consequence of oxidative stress leading to a counter-regulatory upregulation of SOD [74]. In addition, smokers had a lower catalase and SOD response to acute exercise, showing that the antioxidant defense system is not primed to respond to a sudden increase in free radicals [57]. In general, it is not clear if the upregulation or downregulation of antioxidant enzymes is positive or negative signal, but it is clear that smoking impairs the response and the ability of the antioxidant defense system to address acute and chronic oxidative stress.

## Inflammation and lipid metabolism

It is evident that oxidative stress causes ED by interfering with ^•^NO signaling. Inflammation and oxidative stress are tightly bound, when it comes to ED, as oxidative stress-caused pro-inflammatory processes in the endothelial cells signal immune cells to infiltrate the endothelium. This again leads to a localized production of free radicals, mostly macrophage-derived superoxide. The infiltrated immune cells then accumulate oxidized lipids to become foam cells and form atherosclerotic plaques. This not only interferes with vascular homeostatic signaling chemically (via production of free radicals), but also physically through separation of the endothelium from smooth muscle cells, and, at later stages, by disrupting the endothelial cell monolayer [25, 52]. In the 2004 edition of the Report of the Surgeon General on the health consequences of smoking, there is a strong emphasis on inflammation and lipid metabolism alterations [76], as the endothelium plays an important role in anti-inflammatory and anti-thrombotic response as well. It was observed that smokers have an elevated level of circulating markers of inflammation like C-reactive protein and interleukin-6 [7, 75], and also increased levels of VCAM-1 and ICAM-1, which indicates higher adhesion of immune cells to the endothelium [51]. Although data from human studies on acute smoking are not consistent, animal studies showed a stable increase in circulating immune cells and inflammatory markers, such as TNF-α [77]. This was also confirmed by results from cell culture experiments [41, 69]. Previous tobacco cigarette research revealed that smokers have a higher circulating low density lipoprotein (LDL), and lower circulating high density lipoprotein (HDL) cholesterol [16, 22]. As tobacco smoking increased oxidative stress leads to more products of lipid peroxidation [33, 65], more oxidized LDL will likely be present in the circulation of smokers. Indeed, experiments did reveal that cigarette smoke can oxidize LDL, both in vitro [23] and in vivo [81]. In addition, it is known that oxidatively modified LDL enhances monocyte adhesion to the endothelium and progression of atherosclerosis [78]. Animals treated with cigarette smoke extract injections or being exposed to second hand smoke exhibited increased oxidative modifications of LDL [24, 80]. Importantly, smoking cessation remains the most effective way to prevent oxidative stress, lipid peroxidation, and high circulating oxidized LDL [44, 61].

## Vascular (endothelial) dysfunction and tobacco cigarette smoking in epidemiological/observational studies

**Fig. 4:**
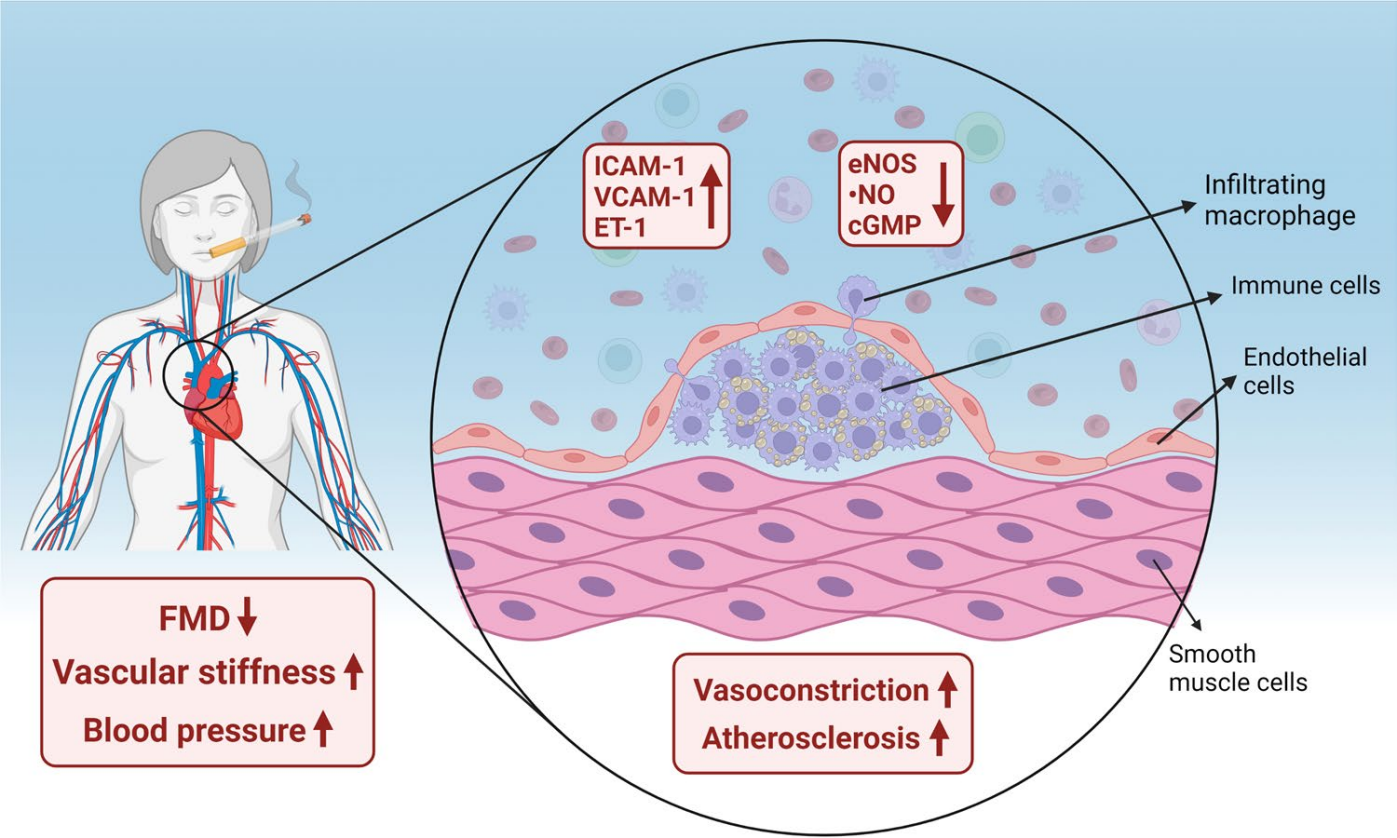
Mechanisms of increased blood pressure in chronic smokers. ED is initiated and propagated by smoking. Clinical studies have demonstrated increased blood pressure and vascular stiffness, and decreased flow-mediated dilation (FMD) in subjects after smoking only one cigarette, but also in chronic smokers. The increase in secreted adhesion molecules (ICAM-1 and VCAM-1) promotes immune cell infiltration into the endothelium. Accumulated immune cells impair vascular function through decrease in nitric oxide (^•^NO) signaling, promoting increased vasoconstriction and future atherosclerosis. Created with BioRender.com

Large-scale epidemiological studies have demonstrated that cigarette smoking is associated with vascular (endothelial) dysfunction. It still remains to be established whether smoking is affecting more endothelial function of arterial conductance or resistance vessels. Recent studies from Gutenberg Health Cohort Study (GHS, *N*=15,010 at baseline) by Omar Hahad et al. demonstrated that tobacco smoking was associated with ED of resistance arteries only (measured by reactive hyperemia index and reflection index), while no association with ED of arterial conduit arteries (measured by FMD, brachial artery) was found [31]. In a subsequent study by Hahad et al., the authors could also demonstrate that arterial stiffness and wave reflection determined by stiffness index and augmentation index were dose-dependently associated with smoking status, pack-years of smoking, and years since quitting smoking based on cross-sectional GHS data [32]. It is important to acknowledge that the early phase of the atherosclerotic process is characterized by ED, whereas the later phases result in arterial stiffness, a marker that also reflects vascular ^•^NO bioavailability and associates with risk of cardiovascular events [55]. Interestingly, even in teenagers, smoking exposure at low levels was shown to be associated with increased arterial stiffness [14]. In 1926 participants from the Ludwigshafen Risk and Cardiovascular Health (LURIC)—a prospective case-control study in patients who underwent coronary angiography, Delgado et al. showed higher concentrations of circulating markers of endothelial function in smokers including sICAM-1, sE-selectin, and sP-selectin, but lower concentrations of sL-selectin and sVCAM-1 compared to never-smokers [18]. In the Circulatory Risk in Communities Study (CIRCS), heavy and chronic smoking were associated with a high prevalence of impaired endothelial function (defined by FMD <5.1% (lowest quartile) and <6.8% (median)), in a cross-sectional analysis of 910 men and women [17]. Langham et al. also could demonstrate that markers of endothelial function assessed by quantitative cardiovascular magnetic resonance in the peripheral and central vasculature are sensitive to smoking [47]. In 2209 Japanese men, an increase in the number of cigarettes smoked (defined by pack-years) was associated with impaired ED as evidenced by decreased FMD [34]. In addition, in a sample of autosomal dominant polycystic kidney disease patients with preserved renal function, FMD was significantly lower in smoking compared to non-smoking patients [27]. A summary of the observed effects is presented in Fig. 4. In addition, smoking cessation has been shown in a prospective randomized trial to strikingly improve flow-dependent dilation [40].

## Conclusions and clinical implications

Taken together, there is strong evidence from human studies that tobacco cigarette smoke exposure has severe cardiovascular side effects leading to ED, increased oxidative stress, inflammation, and ultimately to increased cardiovascular morbidity and mortality. However, ED can be seen as a convergence point for the majority of these smoking-induced pathophysiological mechanisms. Although good research pointing to tobacco toxicity exists for more than 70 years, it is still not clear, which specific chemical compounds are most responsible for the multiple side effects observed in the cardiovascular system. Vascular signaling is disturbed in response to smoking by a complex interplay of enzymatic and small molecule interactions that can be influenced by many factors. Although a complete ban of smoking would be preferable from a clinical point of view (as introduced in New Zealand (https://www.theguardian.com/world/2022/dec/13/new-zealand-passes-world-first-tobacco-law-to-ban-smoking-by-2025)), it will be unrealistic to achieve this kind of prohibition in Germany or other European countries. Therefore, high-quality mechanistic studies of smoking-induced cardiovascular disease are highly needed to further understand the pathomechanism of tobacco smoking for better preventive measures to protect the most vulnerable groups. Finally, future efforts should also focus on (emerging) trends such as cannabis smoking and e-cigarette use in the light of ED and CVD risk.
